# Factors Influencing Prognosis After Initial Inadequate Excision (IIE) for Soft Tissue Sarcoma

**DOI:** 10.1080/13577140310001650321

**Published:** 2003

**Authors:** Albert N. Van Geel, Alexander M. M. Eggermont, Patrick E. J. Hanssens, Paul I. M. Schmitz

**Affiliations:** 1 Department of Surgical Oncology University Hospital Rotterdam/Daniel den Hoed Cancer Center Groene Hilledijk 301 Rotterdam 3075 EA The Netherlands; 2 Department of Radiotherapy University Hospital Rotterdam/Daniel den Hoed Cancer Center Rotterdam The Netherlands; 3 Department of Trials and Statistics University Hospital Rotterdam/Daniel den Hoed Cancer Center Rotterdam The Netherlands

## Abstract

*Purpose*. The influence of initial inadequate excision (IIE) of soft tissue sarcoma (STS) on local control and overall survival is not well established. It is generally believed that an IIE may have a negative impact on both, despite subsequent treatment by radical surgery and radiotherapy. However, data on local recurrence-free survival/overall survival are conflicting and there are no data on the effect of IIE on overall survival.

*Patients and methods*. A retrospective analysis was made of 86 patients with soft tissue sarcoma of the extremities and trunk after an IIE had been performed due to inappropriate work-up. The minimal follow-up was 7 years. Specimens of the subsequent radical resection were evaluated for residual tumor, grade of tumor and complications of IIE. Endpoints were recurrence-free survival and overall survival.

*Results*. Specimens of the subsequent radical resection showed residual tumor in 66 patients (77%). The most common complication after IIE was hematoma. In both univariate and multivariate analyses, grade II/III tumors and complications after IIE are significant negative prognostic factors for local recurrence-free survival (*P* = 0.008 and *P* = 0.002, respectively, in the Cox model). For this survival, three prognostic groups could be formed based on grade, or presence or absence of complications. Adjuvant radiotherapy did not change the rate of local recurrence-free survival. For overall survival, only tumor grade is a significant factor (log-rank test).

*Conclusion*. This retrospective study shows that complications associated with an IIE have a significant negative effect on local control, but not on overall survival, because IIE is often the result of inappropriate work-up before surgery. For better diagnosis and therapy STS should be treated in specialized centers.


*Sarcoma*, September/December 2003, *VOL. 7, NO. 3/4, 159–165*

Taylor & Francis healthsciences

ORIGINAL ARTICLE

### Factors influencing prognosis after initial inadequate excision (IIE) for soft tissue sarcoma

ALBERT N. VAN GEEL[1], ALEXANDER M.M. EGGERMONT[1], PATRICK E.J. HANSSENS[2] & PAUL I.M. SCHMITZ[3]

*Departments of [1]Surgical Oncology, [2]Radiotherapy and [3]Trials and Statistics, University Hospital Rotterdam/Daniel den Hoed Cancer Center, Rotterdam, The Netherlands*

**Abstract**

*Purpose.* The influence of initial inadequate excision (IIE) of soft tissue sarcoma (STS) on local control and overall survival is not well established. It is generally believed that an IIE may have a negative impact on both, despite subsequent treatment by radical surgery and radiotherapy. However, data on local recurrence-free survival/overall survival are conflicting and there are no data on the effect of IIE on overall survival.

*Patients and methods.* A retrospective analysis was made of 86 patients with soft tissue sarcoma of the extremities and trunk after an IIE had been performed due to inappropriate work-up. The minimal follow-up was 7 years. Specimens of the subsequent radical resection were evaluated for residual tumor, grade of tumor and complications of IIE. Endpoints were recurrence-free survival and overall survival.

*Results.* Specimens of the subsequent radical resection showed residual tumor in 66 patients (77%). The most common complication after IIE was hematoma. In both univariate and multivariate analyses, grade II/III tumors and complications after IIE are significant negative prognostic factors for local recurrence-free survival ($P = 0.008$ and $P = 0.002$, respectively, in the Cox model). For this survival, three prognostic groups could be formed based on grade, or presence or absence of complications. Adjuvant radiotherapy did not change the rate of local recurrence-free survival. For overall survival, only tumor grade is a significant factor (log-rank test).

*Conclusion.* This retrospective study shows that complications associated with an IIE have a significant negative effect on local control, but not on overall survival, because IIE is often the result of inappropriate work-up before surgery. For better diagnosis and therapy STS should be treated in specialized centers.

**Key words:** *soft tissue sarcoma, surgery, prognostic factors, local recurrence, survival*

### Introduction

Soft tissue sarcoma (STS) is a group of anatomical and histological diverse malignancies of mesenchymal tissue. STS is a rare condition: in the USA there are about 7000 new cases and 4300 deaths each year.[1]

Local control of STS mainly depends on the extent of surgical treatment. The most effective local control is achieved by resection of the tumor with clear margins; however, the definition of radical resection is still debated. The definition varies from wide resection to compartment resection and myectomy, with less radical resections being defined as marginal or intralesional ones. The local recurrence rate of these latter resections ranges from 60 to 95%, whereas the local recurrence rate of radical resection ranges from 5 to 30%.[2–4] When a radical resection cannot be achieved, radiotherapy can improve the local control rate, but will rarely match the better rate of radical resections. Improved local control rate is not reflected in a better disease-specific survival.[5] Brachytherapy is reported to achieve better local control than conventional radiotherapy, especially for high-grade sarcomas.[6]

Other factors influencing the local control rate are tumor size, depth, retroperitoneal and visceral localizations, and histological grade.[7]

In addition, factors such as age or specific histological types have occasionally been reported.[8,9]

In practice, a substantial number of patients undergo a marginal resection because the tumor

Correspondence to: A.N. van Geel, MD, PhD, Department of Surgical Oncology, Erasmus Medical Center—Daniel den Hoed Cancer Center, Groene Hilledijk 301, 3075 EA Rotterdam, The Netherlands. Tel.: +31-10-4391793; Fax: +31-10-4391011; E-mail: geel@chih.azr.nl

1357-714X print/1369–1643 online © 2003 Taylor & Francis Ltd
DOI: 10.1080/13577140310001650321

has not been recognized as a STS. After the pathological diagnosis of STS is established, a subsequent treatment must be performed, preferably by a radical resection whether or not in combination with radiotherapy. There are few data on the effect of these unplanned initial inadequate resections (IIE) with no clear margins on local recurrence rate,[9–11] and no data at all on survival. This issue also has been studied, with conflicting results, only in patients with an incisional biopsy, a problem that may be closely related to an IIE.[3,12]

### Patients and methods

All records of 263 patients with STS referred for surgery to the University Hospital Rotterdam/Daniel den Hoed Cancer Center between 1986 and 1993 were reviewed. Excluded from this study were those patients with a local recurrence and/or distant metastases at time of referral. Retrospectively, those patients were identified who had a previous irradical resection for diagnostic reasons, which could not be classified as an incisional biopsy. Only patients with STS in the limbs, girdles and trunk were entered in this study because, in these patients, a radical treatment (radical resection, marginal resection with radiotherapy or amputation) is always possible. Small superficial tumors ($\leq 5$ cm) were also excluded.

Pathological examination was carried out in our hospital, on all resected specimens of all patients, also when the initial surgery was performed in another hospital. Excluded from this study were those pathological subtypes of STS that need a non-surgical approach as the first-line treatment (e.g., small round-cell sarcomas such as Ewing sarcoma), or subtypes with a specific biological behavior such as borderline tumors (e.g., aggressive fibromatosis and desmoids), because they do not give distant metastases.

For statistical reasons it was decided to include only patients treated before 1993, thus assuring a follow-up period of at least 7 years in all patients.

In our records we found 86 patients (32.7% of all patients referred for treatment) fulfilling the above-mentioned criteria. The following data were collected: age at the time of diagnosis, tumor site, date and type of initial surgery (excision or debulking), complications of this procedure (hematoma, infection), type of incision, residual tumor seen on CT or MRI after initial surgery, date and type of subsequent definitive surgery, whether definitive surgery was preceded by an isolated limb perfusion, and whether definitive surgery was followed by adjuvant radiotherapy and/or adjuvant chemotherapy. Histopathological work-up included: whether a macro- or microscopical residual tumor was seen at pathological examination, total diameter of the tumor, including the size of the IIE, and pathological type and grade of tumor. Furthermore, the complexity of the definitive surgical procedure was evaluated compared with the one surgical procedure that would have been performed after an initial appropriate preoperative work-up. Finally, data on morbidity, local control and survival were noted.

### Statistical analysis

The potential prognostic factors underwent univariate analysis with respect to three endpoints: time to death, time to local recurrence and time to distant metastases. Local recurrence-free survival was calculated for those patients alive at 2, 5 and 10 years. Log-rank tests and Kaplan-Meier survival analyses were used for all factors and endpoint combinations for all 86 studied patients. In multivariate analysis, only local control was studied using the Cox proportional hazards model. Based on the results of the multivariate analysis we formed three prognostic groups (good, moderate and poor prognosis).

### Results

In this series of 86 patients, there were 47 males and 39 females (age range 17–77 years). The tumor was located in the lower limb in 39 cases (45%), the upper limb in 17 (20%), the trunk in 19 (22%) and in the girdles in 11 (13%). There were 21 superficial tumors larger than 5 cm and 65 deep, subfacial tumors.

In 51 patients (60%), the operation was considered to be an excisional biopsy. In the remaining 35 patients (40%), only a gross debulking of the tumor was performed.

At presentation in our hospital, a hematoma was seen in 20 (23%) of the 86 patients. In three patients, a wound infection at presentation needed immediate drainage, and another three patients were treated by antibiotics. Transverse scars were seen in 15 patients.

Within 3–4 weeks after referral, a CT scan or MRI was performed for local staging. In 40 patients (46%), no residual tumor could be found. In the remaining 46 patients (54%), there were changes in the surgical field that could not be explained by the previous surgery alone, with a strong suspicion of macroscopic tumor remnant being present. In the resected specimens, residual tumor was found in 66 patients (77%).

On pathological examination, liposarcoma was the most common subtype and was found in 17 patients (20%), all other types represented less than 10% of the STS.

The tumor was graded according to Trojani as follows: grade I in 28 patients (33%), grade II in 44 (51%) and grade III in 14 (16%).

The diameter of the tumor was: T1 ($\leq 5$ cm) in 41 patients and T2 ($> 5$ cm) in 45 patients.

In the absence of distant metastases, all patients underwent a radical treatment for their primary lesion: whenever feasible, a radical resection was performed ($n = 45$) and in other cases a less radical resection with adjuvant radiotherapy ($n = 33$). In our institute, a radical resection is defined as a resection of the tumor with a free margin of at least 1 cm at pathological examination, or less than 1 cm when, at that margin, fascia, periostium or perichondrium is present. Four patients did not receive further treatment after IIE and another four patients received only radiotherapy.

Radiotherapy was delivered to the target area (compartment or field round the tumor bed with a margin of 5–7 cm length and 2 cm transverse) at a dosage of 50 Gy in 25 fractions, with a boost on the tumor bed of 10 Gy in five fractions. The total dosage was increased to 70 Gy in cases of limited resection margins.

In five cases, an isolated limb perfusion was the initial treatment in order to prevent amputation or major functional morbidity. Five patients were treated in the context of the EORTC protocol 10863 with neoadjuvant chemotherapy (doxorubicin 50 mg/m$^2$ and ifosfamide 5 mg/m$^2$ i.v.q. 3 weeks, three courses) and were excluded from multivariate analysis for local recurrence rate because they were recognized as high-risk patients.

In 50% of patients, the definitive surgery was considered to be more complex and resulted in more morbidity than could be expected had the tumor been diagnosed and treated in the proper way at first presentation. In 71 patients (82%), the postoperative course was uneventful.

All patients were followed for 7 years or longer. Of the total group, 25 patients (29%) developed a local recurrence and 28 patients (33%) distant metastases; 14 patients (16%) had both local and distant recurrent disease.

At the close of the study, 52 patients (60%) were still alive without any evidence of STS and four patients (5%) were alive with evidence of recurrent tumor. A total of 30 patients (35%) had died, five of whom died from intercurrent diseases.

Table 1 gives data on overall survival, local recurrence-free and distant recurrence-free survival at 2, 5 and 10 years follow-up.

Table 2 lists the prognostic parameters investigated and Table 3 gives the results of the univariate analyses.

**Table 1.** *Survival data of 86 patients with an initial irradical excision for soft tissue sarcoma*

| | Overall survival (%) | Local recurrence-free survival (%) | Distant recurrence-free survival (%) |
|---|---|---|---|
| 2 years | 86 | 86 | 85 |
| 5 years | 75 | 74 | 74 |
| 10 years | 55 | 65 | 56 |

**Table 2.** *Study parameters of 86 patients with an unplanned excision of soft tissue sarcoma*

Potential prognostic factors
1. Age at the date of the first operation
2. Gender
3. Localization of the primary: lower limb, upper limb, trunk (abdominal wall, back, and buttock) and girdles (axilla and groin)
4. Complication after the first operation: hematoma
5. Definitive surgery: radical resection +/· isolation perfusion or amputation, less radical resection +/· isolation perfusion and radiotherapy, no therapy and radiotherapy alone
6. The definitive surgical procedure was more complex than could be expected had proper diagnosis and treatment taken place initially. Also, that adjuvant radiotherapy could be avoided in case of residual macroscopical tumor or microscopical high-grade tumor)
6. Tumor diameter before any treatment
7. Histopathological diagnosis (liposarcoma, all other types of sarcoma)
8. Grade according to Trojani
9. Macroscopical residual tumor seen at CT scan or MRI after initial surgery
10. Microscopical residual tumor found only at histopathological examination
11. Data of follow-up (date of local recurrence and/or distant metastases, last date of follow-up and survival status)

Follow-up Data
1. Survival status and date of death or last date of follow-up
2. Local recurrence status and date of local recurrence (if occurred)
3. Distant metastases status and date of distant metastases (if occurred)

**Table 3.** *Data (P values) on the potential prognostic factors of initial irradical excision of soft tissue sarcoma in 86 patients (significant* p < *0.05)*

| | Overall survival | Local recurrence-free survival | Distant recurrence-free survival |
|---|---|---|---|
| Age at diagnosis | 0.08 | 0.16 | 0.43 |
| Gender | 0.40 | 0.27 | 0.11 |
| Time to symptoms | 0.46 | 0.99 | 0.73 |
| Localization | 0.12 | 0.39 | 0.87 |
| Complication | 0.16 | **0.002** | **0.048** |
| Definitive treatment | 0.54 | **0.006** | 0.07 |
| Complexity | 0.16 | **0.002** | **0.048** |
| Residual tumor | | | |
| Macroscopic | 0.18 | 0.02 | 0.12 |
| Microscopic | 0.31 | 0.17 | 0.32 |
| Diameter | 0.18 | 0.52 | 0.25 |
| Pathology | 0.43 | 0.46 | 0.17 |
| Grade | **0.01** | **0.004** | **0.0003** |

**Table 4.** *Prognostic subgroups of patients with an initial irradical excision of soft tissue sarcoma (*n=*81)*

| | | Local recurrence-free survival (%) | | |
|---|---|---|---|---|
| Group | | 2 years | 5 years | 10 years |
| I (*n* = 23) | Good prognosis | 100 | 100 | 86 |
| II (*n* = 43) | Moderate prognosis | 93 | 79 | 86 |
| III (*n* = 15) | Poor prognosis | 67 | 37 | 37 |

Group I, grade I and no complication; group II, grade II/III or complication; and group III, grade II/III and complication.

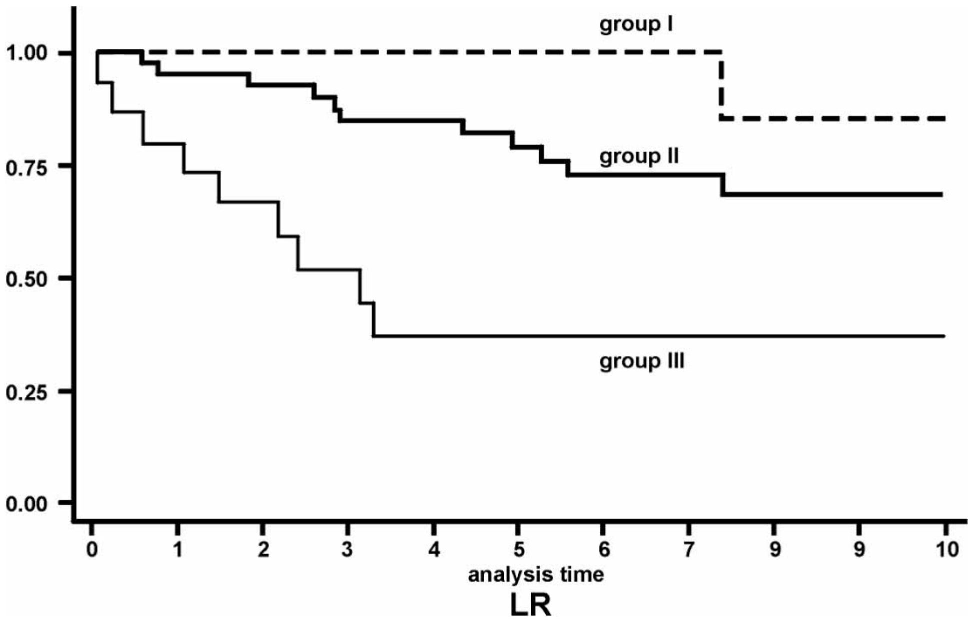

**Fig. 1.** *Local recurrence-free survival curves of 81 patients with initial irradical excision for soft tissue sarcoma: group I, good prognosis (*n = *23); group II, moderate prognosis (*n = *43); group III, poor prognosis (*n = *15).*

The Cox proportional hazards model was used to determine local recurrence-free survival. Only complication ($P = 0.002$) and tumor grade ($P = 0.008$) appeared to be independent prognostic factors for this endpoint. Based on these two factors, three prognostic groups were created. These groups (defined as good, moderate and poor) are described in Table 4 and the local recurrence-free survival is shown in Figure 1.

Table 5 presents data on the local recurrence-free survival for the three prognostic groups, with or without radiotherapy.

**Table 5.** *Local recurrence-free survival for three prognostic subgroups with or without radiotherapy (n = 81)*

| | | Local recurrence-free survival (%) | | |
|---|---|---|---|---|
| | | 2 years | 5 years | 10 years |
| Group without radiotherapy | | | | |
| I ($n = 18$) | Good prognosis | 100 | 100 | 80 |
| II ($n = 21$) | Moderate prognosis | 100 | 94 | 94 |
| III ($n = 6$) | Poor prognosis | 66 | 50 | 50 |
| Group with radiotherapy | | | | |
| I ($n = 5$) | Good prognosis | 100 | 100 | 100 |
| II ($n = 22$) | Moderate prognosis | 86 | 65 | 47 |
| III ($n = 9$) | Poor prognosis | 66 | 26 | – |

Group I, grade I and no complication; group II, grade II/III or complication; and group III, grade II/III and complication.

### Discussion

Resection of STS with inadequate margins is considered the most negative prognostic factor in local recurrence-free survival. As early as 1986, Suit and Tepper concluded that local recurrence rates vary according to the initial surgical procedure, and that improved local control correlates with better survival.[13] However, data from many large studies do not support this conclusion and do not identify local recurrence as an independent unfavorable prognostic factor for survival. In all studies, tumor grade was the most important and dominant prognostic factor.[14,15]

Improvement of preoperative imaging by CT and MRI and improvement of treatment modalities (e.g., surgery alone or in combination with radiotherapy and chemotherapy including regional isolated limb perfusion) have increased the local control rate to about 85%.[16,17]

All patients who have undergone an IIE for STS are candidates for a second operation in order to achieve adequate surgical margins. A poorly performed open biopsy or IIE can change the definitive surgical treatment in at least 30%.[18,19] In the recent literature, we found no data to show that patients with a radical resection as first treatment will have a better local recurrence-free survival than those with a radical resection after IIE (so-called ‘whoops operation’). Nor could we find factors in the latter group with an adverse effect on overall survival.

In some series, the systematic use of an incisional biopsy is reported to be the cause of higher local recurrence rates.[20] However, Collin *et al.* could not confirm this statement.[12] Of note is a recent study from the Memorial Sloan-Kettering Cancer Center, which reports better overall survival in patients with re-resection after previous resection elsewhere, compared with one definitive resection in the Cancer Center as primary treatment; this is especially significant for high-grade STS.[21] The authors then concluded that, where possible, re-resection should be liberally applied in patients with primary extremity STS.

For the reasons mentioned above, we studied a group of patients who had undergone an IIE elsewhere and who were referred to our center for definitive treatment.

After 5 years, the overall survival was 75%, the local recurrence-free survival was 74% and the distant recurrence-free survival was 74%, these percentages correspond with data in literature.

Two retrospective studies, similar to ours, also reported the problem of unplanned excision. In 1996, Goodlad *et al.* studied 95 patients but did not report morbidity and follow-up data,[10] and Noria *et al.* investigated 65 patients but included only extremity sarcomas without evidence of macroscopical tumor before second surgery.[11] We found residual disease in 66 patients (77%), which is higher than reported in the latter two series, i.e., 59 and 35%, respectively. In 20 of the 66 patients in our series and in 25 of the 56 patients in the Goodlad *et al.* series, the residual disease was found only at pathological examination. In another study by Giuliano and Eilber, the number of patients with residual bulky disease was the same as those with residual disease found only at pathology.[22]

Because these earlier studies give no information on the postoperative course and complications after IIE no comparison can be made with our results, nor was any information given about the influence of the first operation on the extent of the definitive resection and or the number of patients who subsequently received adjuvant radiotherapy.

When comparing the definitive surgical procedure with the one procedure that might have been performed after an appropriate preoperative work-up we realize that ‘more complex’ is a subjective criterion. Therefore, we give an example to explain what is intended by ‘more complex surgery’: for instance, a transverse scar in the extremity can lead to difficulty in resecting the scar and the former surgical field and a skin graft may be used to avoid too much traction; Mankin *et al.* also addressed this problem.[23] Inappropriate placement of the biopsy scar conflicts with the definitive resection. The surgeon involved in the definitive treatment should also perform the biopsy, and this may explain the better outcomes in specialized centers.[24] The group of patients studied by Tovnik with high grade deep-seated sarcomas referred with untouched lesions, had a local recurrence rate of 25% compared with 33% for those referred after biopsy or incomplete excision ($P = 0.2$).[25] In these patients there was also a tendency towards more inadequate final margins, but this was counteracted by increased use of radiotherapy. Adjuvant radiotherapy can introduce co-morbidity, especially in the neighborhood of the joints. However, in our three subgroups of patients we cannot conclude that radiotherapy contributes to a better local recurrence-free survival.

Open biopsy has been associated with a higher rate of local recurrence (36%) compared to needle or no biopsy (24%, $P = 0.04$).[25]

In our series, 43 of the patients (50%) were subjected to a more complex surgical procedure due to subsequent treatment; this in contrast to initially considering the extent of primary local tumor on MRI or CT followed by a proper surgical treatment, as proposed by our national guidelines.[26]

The data from this analysis suggest that postoperative complications of IIE significantly increase the rate of local recurrence; however, this increased rate does not influence overall survival. This supports the hypothesis that local recurrence is an independent endpoint not related to survival, as reported in recent large studies,[14,15,22] which is in contrast to the first report dealing with this subject.[27] In our series, 63 patients had no complications and 20 patients (23.2%) suffered from a hematoma.

### Conclusions

Our retrospective study does not indicate that IIE has a negative impact on overall survival. With respect to local recurrence, it is shown that 19% of the 86 patients had a bad prognosis due to the grade of the tumor and complications after IIE. These findings are similar to those reported by the Scandinavian Sarcoma Group: inadequate surgical margins contribute to a higher local recurrence rate but not to a higher rate of distant metastases.[27] In STS, tumor grade is a factor, which cannot be influenced, whereas complications after IIE can be avoided because they are related to the surgery. This situation can be improved by systematically referring patients with STS to sarcoma units where a proper invasive diagnostic procedure can be done after adequate imaging. In almost all cases, even an incisional biopsy can be avoided by performing a thru-cut biopsy or fine needle aspiration.[28] In those cases where an incisional biopsy cannot be avoided, the procedure should be performed under strict conditions and according to well-described guidelines.[18,23,29]

In a recent review of the literature, Spiro *et al.* concluded that negative surgical margins achieve the best local control. Less important are other factors such as tumor size, pathological grade, histopathology and concomitant chemotherapy.[31] The (unplanned) initial irradical resection should be added to this list.

### Acknowledgment

Thanks to Mrs Laraine Visser for preparing the English manuscript.

### References

1. *Cancer facts and figures*. Atlanta GA: American Cancer Society, 1999.
2. Shiu MH, Castro EB, Hajdu SI, Fortner JC. Surgical treatment of 297 soft tissue sarcomas of the lower extremity. *Ann Surg* 1975; 182: 597–602.
3. Markhede G, Angervall L, Stener B. A multivariate analysis of the prognosis after surgical treatment of malignant soft tissue tumors. *Cancer* 1982; 9: 1721–33.
4. Rydholm A, Rööser B. Surgical margins for soft tissue sarcoma. *J Bone Joint Surg* 1987; 69A: 1074–8.
5. Rosenberg SA, Tepper J, Glatstein E, Costa J, Baker A, Brennan MJ, DeMoss EV, Seipp C, Sindelar WF, Sugarbaker P, Wesley R. The treatment of soft tissue sarcomas of the extremities: prospective randomized evaluations of (1) limb sparing surgery plus radiation therapy compared with amputation and (2) the role of adjuvant chemotherapy. *Ann Surg* 1982; 196: 305–15.
6. Pisters PWT, Harrison LB, Leung DHY, Woodruff JM, Casper ES, Brennan MF. Long-term results of a prospective randomized trial of adjuvant brachytherapy in soft tissue sarcoma. *J Clin Oncol* 1996; 14: 859–68.
7. Lewis JJ, Brennan MF. Soft tissue sarcomas. *Curr Prob Surg* 1996; 33: 817–72.
8. Coindre JM, Terrier P, Bui N, Barichou F, Collin F, Le Doussal V, Mandard AM, Vilain MO, Jacquemier J, Duplay H, Sastre X, Barlier C, Henri-Amar M, Mace-Lesech J, Contesso G. Prognostic factors in adult patients with locally controlled soft tissue sarcoma; a study of 546 patients from the French Federation of Cancer Centers Sarcoma Group. *J Clin Oncol* 1996; 14: 869–77.
9. Pisters PWT, Leung DHY, Woodruff J, Shiu W, Brennan MF. Analysis of prognostic factors in 1041 patients with localized soft tissue sarcomas of the extremities. *J Clin Oncol* 1996; 14: 1679–89.
10. Goodlad JR, Fletcher CD, Smith MA. Surgical resection of primary soft-tissue sarcoma. Incidence of residual tumour in 95 patients needing re-excision after local resection. *J Bone Joint Surg Br* 1996; 78: 658–61.
11. Noria S, Davis A, Kandel R, Levesque J, O'Sullivan B, Wunder J, Bell R. Residual disease following unplanned excision of soft-tissue sarcoma of an extremity. *J Bone Joint Surg Am* 1996; 78: 650–655.

12. Collin C, Hajdu SI, Godbold J, Shiu MH, Hilaris BI, Brennan MF. Localized, operable soft tissue sarcoma of the lower extremity. *Arch Surg* 1986; 121: 1425–33.
13. Suit HD, Tepper JE. Impact of improved local control on survival in patients with soft tissue sarcoma. *Int J Radiat Oncol Biol Phys* 1986; 12: 699–700.
14. Stotter AT, A'Hearn RP, Fisher C. The influence of local recurrence of extremity soft tissue sarcoma on metastases and survival. *Cancer* 1990; 65: 1119–29.
15. Gustafson P, Rooser B, Rydholm A. Is local recurrence of minor importance for metastases in soft tissue sarcoma? *Cancer* 1991; 67: 2083–6.
16. Eilber FR, Guiliano AE, Huth JF, Morton DL. A randomized prospective trial using postoperative adjuvant chemotherapy (adriamycin) in high grade extremity soft tissue sarcoma. *Am J Clin Oncol* 1988; 11: 39–45.
17. Eggermont AMM, Schrafford Koops H, Klausner JM, Kroon BBR, Schlag PM, Lienard D, van Geel AN, Hoekstra HJ, Meller I, Nieweg OE, Kettelhack C, Ben-ari G, Pector JC, Lejeune FJ. Isolated limb perfusion with tumor necrosis factor and melphalan for limb salvage in 186 patients with advanced soft tissue extremity sarcoma. *Ann Surg* 1996; 224: 756–65.
18. Simon MA. Current concepts review: biopsy of muculoskeletal tumors. *J Bone Joint Surg Am* 1982; 64: 1253–7.
19. Mankin HJ, Lange TA, Spanier SS. The hazards of biopsy in patients with malignant bone and soft tissue tumors. *J Bone Joint Surg Am* 1982; 64A: 1121–7.
20. Rydholm A, Gustafson P, Roöser B, Willen H, Akerman M, Herlin K, Alvegard T. Limb sparing surgery without radiotherapy based on anatomic location of soft tissue sarcoma. *J Clin Oncol* 1991; 9: 1757–65.
21. Lewis JJ, Leung D, Espat J, Woodruff JM, Brennan MF. The effect of re-resection in extremity soft tissue sarcoma. *Sarcoma* 2001; 5: 41.
22. Giuliano AE, Eilber FR. The rationale for planned reoperation after unplanned total excision of soft tissue sarcoma. *J Clin Oncol* 1985; 3: 1344–8.
23. Mankin HJ, Mankin CJ, Simon MA. The hazards of the biopsy, revisted. *J Bone Joint Surg* 1996; 78: 656–63.
24. Yasko AW, Reece GP, Gilles TA, Pollock RE. Limb-salvage strategies to optimalize quality of life: the M.D. Anderson Cancer Center experience. *CA Cancer J Clin* 1997; 47: 226–38.
25. Tovnik CS. Local recurrence of soft tissue sarcoma. A Scandinavian Sarcoma Group project. Primary treatment and local recurrence. *Acta Orthop Scand* 2001; 72(Suppl 300): 13–6.
26. van Geel AN, van Unnik JAM, Keus R. Diagnosis and treatment of soft tissue tumours: the Dutch nationwide accepted consensus. *Sarcoma* 1998; 2: 183–91.
27. Trovnik CS, Bauer HCF, Alvegäärd TA, Anderson H, Blomquist C, Berlin Ö, Gustafson P, Saeter G, Wallöe A. Surgical margin, local recurrence and metastases in soft tissue sarcoma: 559 surgically treated patients from the Scandinavian Sarcoma Group Register. *Eur J Cancer* 2000; 36: 710–6.
28. Heslin M, Lewis J, Woodruff J, Brennan M. Core needle biopsy for diagnosis of extremity soft tissue sarcoma. *Ann Surg Oncol* 1997; 4: 425–31.
29. Hoeber I, Spillane AJ, Fisher C, Meiron Thomas J. Accuracy of biopsy techniques for limb and girdle soft tissue tumours. *Ann Surg Oncol* 2001; 8: 80–7.
30. Spiro IJ, Gebhardt MC, Jennings LC, Mankin HJ, Harmon DC, Suit HD. Prognostic factors for local control of sarcomas of the soft tissues managed by radiation and surgery. *Semin Oncol* 1997; 24: 540–6.

## References

[PDF_00592] Beck M., Woo A., Leunig M., Ganz R. (2001). Gluteus minimus-induced femoral head deformation in dysplasia of the hip.. Acta Orthop Scand.

[PDF_00520] Coindre J. M., Terrier P., Bui N. B., Bonichon F., Collin F., Le Doussal V., Mandard A. M., Vilain M. O., Jacquemier J., Duplay H. (1996). Prognostic factors in adult patients with locally controlled soft tissue sarcoma. A study of 546 patients from the French Federation of Cancer Centers Sarcoma Group.. J Clin Oncol.

[PDF_00542] Collin C., Hadju S. I., Godbold J., Shiu M. H., Hilaris B. I., Brennan M. F. (1986). Localized, operable soft tissue sarcoma of the lower extremity.. Arch Surg.

[PDF_00560] Eggermont A. M., Schraffordt Koops H., Klausner J. M., Kroon B. B., Schlag P. M., Liénard D., van Geel A. N., Hoekstra H. J., Meller I., Nieweg O. E. (1996). Isolated limb perfusion with tumor necrosis factor and melphalan for limb salvage in 186 patients with locally advanced soft tissue extremity sarcomas. The cumulative multicenter European experience.. Ann Surg.

[PDF_00555] Eilber F. R., Giuliano A. E., Huth J. F., Morton D. L. (1988). A randomized prospective trial using postoperative adjuvant chemotherapy (adriamycin) in high-grade extremity soft-tissue sarcoma.. Am J Clin Oncol.

[PDF_00582] Giuliano A. E., Eilber F. R. (1985). The rationale for planned reoperation after unplanned total excision of soft-tissue sarcomas.. J Clin Oncol.

[PDF_00532] Goodlad J. R., Fletcher C. D., Smith M. A. (1996). Surgical resection of primary soft-tissue sarcoma. Incidence of residual tumour in 95 patients needing re-excision after local resection.. J Bone Joint Surg Br.

[PDF_00552] Gustafson P., Röser B., Rydholm A. (1991). Is local recurrence of minor importance for metastases in soft tissue sarcoma?. Cancer.

[PDF_00606] Heslin M. J., Lewis J. J., Woodruff J. M., Brennan M. F. (1997). Core needle biopsy for diagnosis of extremity soft tissue sarcoma.. Ann Surg Oncol.

[PDF_00609] Hoeber I., Spillane A. J., Fisher C., Thomas J. M. (2001). Accuracy of biopsy techniques for limb and limb girdle soft tissue tumors.. Ann Surg Oncol.

[PDF_00518] Lewis J. J., Brennan M. F. (1996). Soft tissue sarcomas.. Curr Probl Surg.

[PDF_00571] Mankin H. J., Lange T. A., Spanier S. S. (1982). The hazards of biopsy in patients with malignant primary bone and soft-tissue tumors.. J Bone Joint Surg Am.

[PDF_00585] Mankin H. J., Mankin C. J., Simon M. A. (1996). The hazards of the biopsy, revisited. Members of the Musculoskeletal Tumor Society.. J Bone Joint Surg Am.

[PDF_00499] Markhede G., Angervall L., Stener B. (1982). A multivariate analysis of the prognosis after surgical treatment of malignant soft-tissue tumors.. Cancer.

[PDF_00537] Noria S., Davis A., Kandel R., Levesque J., O'Sullivan B., Wunder J., Bell R. (1996). Residual disease following unplanned excision of soft-tissue sarcoma of an extremity.. J Bone Joint Surg Am.

[PDF_00513] Pisters P. W., Harrison L. B., Leung D. H., Woodruff J. M., Casper E. S., Brennan M. F. (1996). Long-term results of a prospective randomized trial of adjuvant brachytherapy in soft tissue sarcoma.. J Clin Oncol.

[PDF_00528] Pisters P. W., Leung D. H., Woodruff J., Shi W., Brennan M. F. (1996). Analysis of prognostic factors in 1,041 patients with localized soft tissue sarcomas of the extremities.. J Clin Oncol.

[PDF_00505] Rosenberg S. A., Tepper J., Glatstein E., Costa J., Baker A., Brennan M., DeMoss E. V., Seipp C., Sindelar W. F., Sugarbaker P. (1982). The treatment of soft-tissue sarcomas of the extremities: prospective randomized evaluations of (1) limb-sparing surgery plus radiation therapy compared with amputation and (2) the role of adjuvant chemotherapy.. Ann Surg.

[PDF_00574] Rydholm A., Gustafson P., Röser B., Willén H., Akerman M., Herrlin K., Alvegård T. (1991). Limb-sparing surgery without radiotherapy based on anatomic location of soft tissue sarcoma.. J Clin Oncol.

[PDF_00503] Rydholm A., Röser B. (1987). Surgical margins for soft-tissue sarcoma.. J Bone Joint Surg Am.

[PDF_00496] Shiu M. H., Castro E. B., Hajdu S. I., Fortner J. G. (1975). Surgical treatment of 297 soft tissue sarcomas of the lower extremity.. Ann Surg.

[PDF_00568] Simon M. A. (1982). Biopsy of musculoskeletal tumors.. J Bone Joint Surg Am.

[PDF_00612] Spiro I. J., Gebhardt M. C., Jennings L. C., Mankin H. J., Harmon D. C., Suit H. D. (1997). Prognostic factors for local control of sarcomas of the soft tissues managed by radiation and surgery.. Semin Oncol.

[PDF_00549] Stotter A. T., A'Hern R. P., Fisher C., Mott A. F., Fallowfield M. E., Westbury G. (1990). The influence of local recurrence of extremity soft tissue sarcoma on metastasis and survival.. Cancer.

[PDF_00546] Suit H. D., Tepper J. E. (1986). Impact of improved local control on survival in patients with soft tissue sarcoma.. Int J Radiat Oncol Biol Phys.

[PDF_00600] Trovik C. S., Bauer H. C., Alvegård T. A., Anderson H., Blomqvist C., Berlin O., Gustafson P., Saeter G., Wallöe A. (2000). Surgical margins, local recurrence and metastasis in soft tissue sarcomas: 559 surgically-treated patients from the Scandinavian Sarcoma Group Register.. Eur J Cancer.

[PDF_00596] Van Geel A. N., Van Unnik J. A., Keus R. B. (1998). Diagnosis and treatment of soft tissue tumours: the dutch nationwide-accepted consensus.. Sarcoma.

[PDF_00588] Yasko A. W., Reece G. P., Gillis T. A., Pollock R. E. (1997). Limb-salvage strategies to optimize quality of life: the M.D. Anderson Cancer Center experience.. CA Cancer J Clin.

